# Cultural adaptation to Bolivian Quechua and psychometric analysis of the Patient Health Questionnaire PHQ-9

**DOI:** 10.1186/s12889-023-17566-8

**Published:** 2024-01-09

**Authors:** Juan Carlos Bazo-Alvarez, Adriana Rocío Ortiz Aparicio, Rodrigo Robles-Mariños, Félix Julca-Guerrero, Heber Gómez, Oscar Bazo-Alvarez, Julio Cjuno

**Affiliations:** 1 Universidad Cesar Vallejo, Escuela de Medicina, Trujillo, Peru; 2 Research Department of Primary Care and Population Health, University College London (UCL), London, UK; 3 Universidad Adventista de Bolivia, Escuela de Psicología, Cochabamba, Bolivia; 4 Programa de Medicina, Universidad Peruana de Ciencias Aplicadas (UPC), Lima, Peru; 5 Universidad Nacional Santiago Antúnez de Mayolo, Huaraz, Peru; 6 Instituto de Investigación, Capacitación y Desarrollo Psicosocial y Educativo PSYCOPERU, Lima, Peru; 7 Ingeniería de Sistemas e Informática, Universidad Tecnológica del Perú, Lima, Peru; 8 School of Medicine, Universidad San Juan Bautista, Lima, Peru; 9 Universidad Peruana Unión, Escuela Profesional de Psicología, Lima, Peru

**Keywords:** Patient health questionnaire, Depression, Depressive symptoms, PHQ-9, Indigenous Peoples (Font: MeSH)

## Abstract

**Objective:**

Cultural adaptation of the Patient Health Questionnaire-PHQ-9 to Bolivian Quechua and analysis of the internal structure validity, reliability, and measurement invariance by sociodemographic variables.

**Methods:**

The PHQ-9 was translated and back-translated (English-Quechua-English) to optimise translation. For the cultural adaptation, experts, and people from the target population (e.g., in focus groups) verified the suitability of the translated PHQ-9. For the psychometric analysis, we performed a Confirmatory Factor Analysis (CFA) to evaluate internal validity, calculated α and ω indices to assess reliability, and performed a Multiple Indicator, Multiple Cause (MIMIC) model for evaluating measurement invariance by sex, age, marital status, educational level and residence. We used standard goodness-of-fit indices to interpret both CFA results.

**Results:**

The experts and focus groups improved the translated PHQ-9, making it clear and culturally equivalent. For the psychometric analysis, we included data from 397 participants, from which 73.3% were female, 33.0% were 18–30 years old, 56.7% reported primary school studies, 63.2% were single, and 62.0% resided in urban areas. In the CFA, the single-factor model showed adequate fit (Comparative Fit Index = 0.983; Tucker-Lewis Index = 0.977; Standardized Root Mean Squared Residual = 0.046; Root Mean Squared Error of Approximation = 0.069), while the reliability was optimal (α = 0.869—0.877; ω = 0.874—0.885). The invariance was confirmed across all sociodemographic variables (Change in Comparative Fit Index (delta) or Root Mean Square Error of Approximation (delta) < 0.01).

**Conclusions:**

The PHQ-9 adapted to Bolivian Quechua offers a valid, reliable and invariant unidimensional measurement across groups by sex, age, marital status, educational level and residence.

## Introduction

Depression is a common mental disorder caused by complex interactions between social, psychological and biological factors [1]. In 2021, depression was present in more than 5% of adults worldwide [2], increasing by 25% during the COVID-19 pandemic [3]. In 2018, a study performed in Bolivia -an Andean country with the second largest Quechua-speaking population in South America- reported 11% mild depression and close to 30% intense or very intense depression in a sample of older adults from an urban area of La Paz [4]. Another study in Peru -a neighbouring country- found that living in Andean regions was a critical factor for increasing depressive symptoms, reporting 9.0% of moderate to severe cases, compared to 5.8% in the non-Andean regions [5]. Of 11 713 participants who lived in the Peruvian Andes, 9.4% presented depressive symptoms during the pandemic [6].

The Patient Health Questionnaire-9 (PHQ-9) is a psychometric instrument designed to assess depressive symptoms according to the criteria of the *Diagnostic and Statistical Manual of Mental Disorders—IV* [7]. Originally written in English [8], it is widely used in clinical practice and international research [9]. It has numerous adaptations in more than 18 languages in 24 countries [10], such as Spanish [11], French [12], Mandarin Chinese [13], Russian [14], German [15], Norwegian [16], Persian [17], Lithuanian [18] and even Kinyarwanda [19]. Although the Spanish version has not been previously evaluated in Bolivia (Spanish is also spoken in this country), the PHQ-8, which omits item 9 of the PHQ-9 [20], has been assessed in low-income people from a heavily indigenous area in La Paz and El Alto (Bolivia). This Spanish version showed good psychometric properties, concluding that the PHQ would be appropriate for screening use in this Andean region [21].

However, there still needs to be a Quechua version of the PHQ for the Bolivian population. Bolivia has the second largest number of Quechua speakers in the world, behind Peru, but it has the highest density of Quechua speakers. According to the National Institute for Statistics, there were 1 837 105 Quechua speakers in 2016, representing 16.4% of the population [22]. People who ethnically self-identify as Quechua may perceive and express depressive symptoms differently from a Spanish speaker, which makes it challenging to use the PHQ-9 in Spanish. The ad hoc solution typically adopted by local users (e.g., clinical psychologists) consists of being assisted by a third bilingual person who translates the questions and responses (for example, a younger relative with a different worldview from the person evaluated). However, this practice is not recommended as it can alter the evaluation, reducing its validity and reliability [23].

Our study focused on a) developing the translation and cultural adaptation of the PHQ-9 into Bolivian Quechua and b) analysing the validity of the internal structure, reliability and measurement invariance (by sociodemographic variables) of the adapted instrument.

## Material and methods

### Design and context

This is a study in which we culturally adapted and validated the Bolivian Quechua version of the PHQ-9 from English [24], and reviewed its psychometric properties. This study was conducted in two phases. In the first phase, we performed direct and reverse translation, after which expert judges verified the adaptation to the cultural and linguistic context of the items, and a focus group of Bolivian Quechua speakers provided feedback on the clarity and comprehensibility of the items. In the second phase, data were collected using the Bolivian Quechua PHQ-9 to verify the internal structure and reliability of the adapted version.

### Cultural adaptation phase

#### Translation

The PHQ-9 in its original version (English) [8] was directly translated into Bolivian Quechua. This was done by two freelance translators, who are native Quechua speakers with advanced English knowledge. After the translation was completed, the two translators and two native-speaking Quechua researchers met to discuss the differences in the translations. Once the discrepancies were resolved and the translations unified, we proceeded with the reverse translation (back to English) [24]. This was done by two translators who had English as their native language and advanced knowledge of Quechua. After the back translations were completed, translators met with two study researchers to verify the back translation along with the first translation, fine-tuned details and gave their thumbs up to the final version in Bolivian Quechua.

#### Cultural adaptation to the Quechua context

A cultural adaptation file was prepared to apply the Delphi method (10.5281/zenodo.10075806) with some open questions for non-common words in Quechua, such as: *"Depression"* and *"Without hope"*. Additionally, we sought to consult the change from *"reading the newspaper" to "listening to the radio"*, the change in the response categories of the PHQ-9 and the relationship between the Quechua PHQ-9 and the DSM-V for the diagnosis of major depression. Those questions were sent via email to two expert reviewers who were Quechua-speaking psychologists and had a minimum of three years of experience caring for patients with depression. One had a master's degree, and the other had a bachelor’s degree in psychology. The interaction between each expert and the research team took place in three rounds of emails and a meeting via Zoom to resolve the suggestions and reach a consensus.

Subsequently, a focus group was organised (via Zoom) to confirm cultural adequation. A Quechua-speaking psychologist expert in qualitative methods moderated the session. The meetings lasted approximately 60 min. In the beginning, it was requested to answer the Quechua PHQ-9 in a shared online version via Google Forms. Then, the moderator invited the participants to give their opinion on the clarity and comprehension of the items in a familiar and straightforward language for the Quechua speaker to trigger an informative dialogue among participants. Seven Quechua-speaking people participated (four women and three men); they were bilingual (Quechua and Spanish speakers) and over 18 years of age.

### Psychometric phase

#### Participants

The final version of the previous phase was applied to a sample of 397 adult men and women over 18 years of age living in urban and rural environments in Cochabamba, Bolivia. All the participants were bilingual Quechua speakers (Quechua and Spanish), with sufficient academic training to read Quechua (e.g., incomplete elementary school as a minimum). Participants were parents organised in primary and secondary schools, fellows in Christian churches, and groups of peasant community associations.

#### Instrument

The PHQ-9 has nine items that correspond to depressive symptoms of the DSM-IV [7]. Response options evoke the frequency of appearance of such symptoms in the last two weeks, considering the following Likert-type scale: 0 = not at all, 1 = several days (1–6 days), 2 = most days (7–11 days), 3 = almost every day (12 days or more) (10.5281/zenodo.10075806). Adding response values, a raw score between 0 and 27 is calculated. The Spanish version of the PHQ-9 has shown adequate validity and reliability in similar settings (e.g., unidimensional measure: CFI = 0.936; RMSEA = 0.089; SRMR = 0.039; α = ω = 0.87) [1].

#### Covariates

These were used to characterise the population and study the measurement invariance of the single-factor model according to age, sex, educational level, marital status and place of residence (rural/urban).

#### Procedure

For data collection, two interviewers were trained to perform a standard application of the instrument to all participants (e.g., evaluation not assisted by any third party). All interviewers were psychology students in their third or fourth year of college. The Bolivian Quechua PHQ-9 was presented in Google Forms to all participants, and data collection began in January and ended in July 2022. Although the test form was digital/online, pollsters collected data using tablets. We programmed the software to detect any response time below or above the group's average by more than 3 min (two standard deviations, calculated in a pilot study). If a person responded too quickly or slowly, the system could identify it, alerting the pollster and suggesting a new application.

#### Statistical analysis

We performed a descriptive analysis of all items (e.g., mean, standard deviation, skewness and kurtosis), A Confirmatory Factor Analysis (CFA) of the single-factor model was performed using a WLSMV (Weighted Least Square Mean and Variance Adjusted) estimator. The single-factor or one-dimensional model was selected given the previous evidence in a similar population [1]. We report the standardised betas of the model and the following goodness of fit indices: the Chi-squared for the model versus the baseline, considering acceptable values < 3; the Comparative Fit Index (CFI), which is adequate when it is > 0.90; the Tucker-Lewis Index (TLI), which is acceptable when it is > 0.90. Likewise, the Standardized Root Mean Squared Residual (SRMR) and the Root Mean Squared Error of Approximation (RMSEA) consider adequate with values ≤ 0.08 [25].

Alternatively, MIMIC (Multiple Indicator, Multiple Cause) models were fitted to assess measurement invariance by age, marital status, and educational level (variables for which multigroup CFA was not feasible). The invariance of the intercepts of the indicators and the mean differences of the latent dimensions were evaluated, all through groups according to the mentioned covariates. Each covariate was assessed separately, comparing each of them two types of models: 1) a saturated version where the covariate explains all the observed items but not the latent dimensions and 2) a version of the model of invariant intercept where the covariate explains all the latent dimensions, but not the items. The adjustment indices indicated above are reported and interpreted as well. In each step, to determine a more restricted model as appropriate, we had to identify a ΔCFI or a ΔRMSEA < 0.01 [26].

Reliability was determined using Cronbach's Alpha [27] and McDonald's Omega [27]. To ensure reproducibility, the main analysis codes are found at: https://github.com/JCBAZO/R-PHQ9-Quechua-Bolivia. All the analyses were done in R Studio version 4.0.4, with the “lavaan” [28], “lavaan.survey” [29], “semTools” [30], “semPlot” [31] and “Psych” [32] packages.

#### Ethics

The Institutional Review Board of the Universidad Peruana Unión reviewed and approved the study with approval number 2022-CE-FCS-UPeU-059; Likewise, it was also approved by the Ethics Committee of the Adventist University of Bolivia based in the city of Cochabamba with approval number 02/2022-UAB. The instrument was applied as a self-report in a virtual format (Google Forms), programmed to present informed consent first, so only those who agreed to participate in the study completed the survey. The original instrument’s copyright owner (Pfizer) authorised its use and adaptation through an email.

## Results

### Cultural adaptation phase

The experts and the research team interacted until they achieved the highest adequacy score for each item concerning the translated PHQ-9 (3 in a range of 0 to 3), highlighting its relevance, representativeness, clarity, and cultural equivalence. Additionally, they provided valuable suggestions that are mentioned below.

Some recommendations contributed to improving the adaptation of the word *“Depression”*, which was finally translated as *“Sunquyki ukhupi ancha llakisqa (phutiska)”* (item 2). For the expression “*Without hope*”, the experts recommended implementing the question as* “manaña sina imapis allin kanqachu”* (item 2). Due to the context, the daily activity *"Read newspaper"* was changed to *"Listen to the radio"* since the latter is the most used source of information in the region (item 7).

The Focus group gave a favourable opinion regarding the clarity and comprehension of the Quechua PHQ-9. In addition, they provided valuable feedback on the points that the experts recommended, helping to shape this adaptation as a clear and appropriate instrument to their cultural context.

The response options (Likert type) of the translated PHQ-9 required careful reflection by the judges and the focus group. In particular, the category "More than half the days" presented clarity problems in its translation. The team ultimately recommended using the expressions *“Mana nijayk´aq”, “Wakin p’unchawkunalla”, “Achka p’unchawkuna”, “Sapa p’unchawkunalla”*, representing the English equivalent to *“Never”, “Some days”, “Several days”, “Almost every day”* respectively.

### Psychometric phase

#### Characteristics of participants

Of 397 Quechua-speaking participants, 73.3% were female, 33.0% were between 18 and 30, 56.7% reported primary school studies, 63.2% were single, and 62.0% lived in urban areas (Table 1).

**Table 1:**
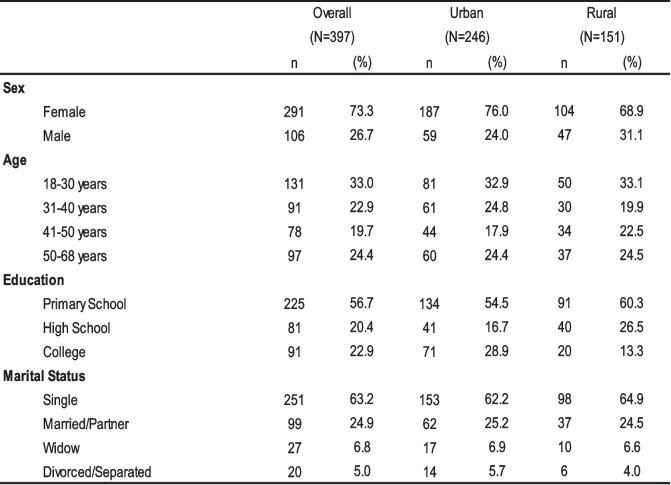
Characteristics of the participants

#### Construct validity

**Fig. 1:**
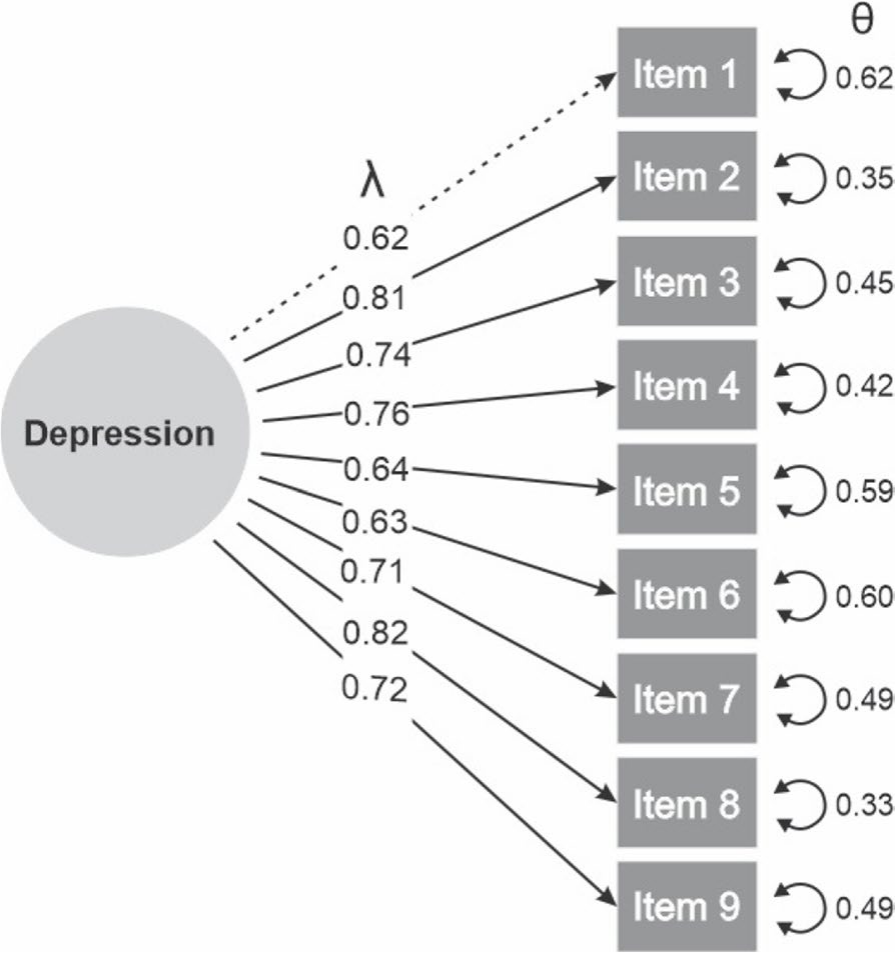
Measurement model of the PHQ-9 with standardized factor loadings and errors (*N* = 397). λ = Lambda of standardized factor loadings; θ = Theta of the residual variance or unique error of a latent variable

The one-dimensional model reported adequate goodness-of-fit values in the overall sample (CFI = 0.983; TLI = 0.977; SRMR = 0.046; RMSEA = 0.069), as well as in urban/rural participants (Table 2). The latent factor of the measurement model (depression) loaded a minimum of λ = 0.62 and a maximum of λ = 0.82 to the PHQ-9 items (Fig. 1).

**Table 2:** Goodness of fit (GOF) of the PHQ-9 measurement model and reliability, overall and by Quechua urban or rural setting

Model	GOF Statistics	Overall (*N* = 397)	Urban (*N* = 246)	Rural (*N* = 151)
1-dimension	X2 (36)	3004	1860	1301
	CFI	0.983	0.976	0.989
	TLI	0.977	0.968	0.986
	SRMR	0.046	0.055	0.057
	RMSEA	0.069	0.081	0.058
	Alpha	0.872	0.869	0.877
	Omega	0.877	0.874	0.885

X^2^(df) = chi-square statistic (χ^2^) with degrees of freedom (df), *CFI* Comparative fit Index, *TLI* Tucker-Lewis index, *SRMR* Standarized root mean squared residual, *RMSEA* Root mean squared error of approximation

#### Reliability

Internal consistency was optimal, showing Cronbach’s Alpha values between 0.869 and 0.877 and Omega’s values between 0.874 and 0.885 (Table 2).

#### Measurement invariance

MIMIC models confirmed the invariance by age, sex, marital status, educational level, and rural/urban settings (Table 3). For all the variables studied, the CFI and TLI values were > 0.96, while the SRMR and RMSEA values were < 0.08. Likewise, the absolute values of ΔCFI, ΔTLI and ΔRMSEA were < 0.01.

**Table 3:** Goodness of fit of the PHQ-9 Multiple-Indicators Multiple-Causes (MIMIC) models (*N* = 397)

**Covariantes**	**Modelo**	**CFI**	**TLI**	**RMSEA**	**SRMR**	**ΔCFI**	**ΔTLI**	**ΔMRSEA**
**Age**	Satured MIMIC	0.979	0.972	0.07	0.049			
	Invariant intercept MIMIC	0.973	0.972	0.069	0.049	-0.006	0.000	-0.001
**Sex**	Satured MIMIC	0.995	0.995	0.041	0.047			
	Invariant intercept MIMIC	0.996	0.995	0.041	0.047	0.001	0.000	0.000
**Marital status**	Satured MIMIC	0.983	0.978	0.068	0.046			
	Invariant intercept MIMIC	0.982	0.982	0.062	0.046	-0.001	0.004	-0.006
**Education**	Satured MIMIC	0.979	0.972	0.07	0.049			
	Invariant intercept MIMIC	0.969	0.968	0.075	0.048	-0.010	-0.004	0.005
**Urban/rual settings**	Satured MIMIC	0.998	0.998	0.025	0.045			
	Invariant intercept MIMIC	0.997	0.996	0.036	0.045	-0.001	-0.002	0.011

*CFI* Comparative fit index, *TLI* Tucker-Lewis index, *SRMR* Standarized root mean squared residual, *RMSEA* Root mean squared error of approximation, Δ difference

## Discussion

This is the first PHQ-9 cultural adaptation to Bolivian Quechua. After performing a two-way translation (English-Quechua-English), we completed the cultural adaptation supported by expert judges and target population representatives, all Quechua speakers. The adapted version offers a unidimensional, reliable and invariant measurement of depressive symptoms across groups by residence, sex, age, marital status, and educational level. Thus, comparisons can be made with the Bolivian Quechua PHQ-9 measurements across the mentioned groups.

This cultural adaptation opens up new possibilities for a more accurate depressive symptoms assessment in the Bolivian Quechua population, either for research or clinical purposes. A narrative review conducted by searching in Pubmed, Web of Science, and Scopus, found seven studies that evaluated depression in Quechua-speaking populations [33]. Only two of these studies used an instrument translated and adapted into Quechua: the Hopkins Symptoms Checklist (HSCL-25). However, this adaptation only considered the Ayacucho-Chanka variant of Quechua [34], differing from the most used in Bolivia, the Cusco-Collao Quechua [35]. Our cultural adaptation of the PHQ-9 corresponds to the latter. In practice, the Ayacucho-Chanka HSCL-25 can be directly applied to a small fraction of the target population. At the same time, for other people, there is still a need to find someone to help clarify the questions and answers to the examiner and examinee. This need for a third party in the evaluation could affect the measurement validity by adding noise from their particular interpretation [23]. The PHQ-9 adapted here reduces the need for such support, facilitating standardised assessment for research (e.g., Demographics and Health Surveys) and clinical practice (e.g., a standardised measurement needed for completing a DSM-V diagnostic).

The adapted PHQ-9 has good psychometric properties, similar to the original and Spanish versions. In both cases, the internal structure of the PHQ-9 was determined as one-dimensional, that is, a single latent factor representing depression, which is expressed by each of the nine symptoms evaluated [1, 8]. Internationally, recent systematic evidence also supports the one-dimensional model across diverse cultures [36], which is consistent with the evidence reported in Peru (Andean country) [1] and our findings in Bolivia. The conclusion of optimal reliability reported here is consistent with the findings from other studies in Spanish speakers -with a similar sociocultural context—such as Peru α = 0.870 and ω = 0.870 [1], Ecuador α = 0.852 and ω = 0.855 [37] or Chile α = 0.891 and ω = 0.896 [38]. This conclusion is even extended to other very different sociocultural contexts, such as Kenya (α = 0.840 and ω = 0.840) [39], showing that the good internal consistency of PHQ-9 is stable across populations.

The Bolivian Quechua PHQ-9 has measurement invariance like other international versions. In Peru, strict invariance was reported for comparisons between sex, age groups, education level, socioeconomic status, marital status, and residence area [1]. In China, invariance across age and sex -including strict invariance—was reported [13]. In Kenya, the configurational, metric and scalar invariance were determined across HIV infection, sex and age [39]. In the United States, the instrument showed invariance when comparing English-speaking and Spanish-speaking women [40] and college students by age and race [41]. In Norway, invariance was reported according to women's presence or absence of eating disorders [16]. A recent systematic review confirmed measurement invariance in at least 18 types of groups, including those determined by the sociodemographic variables included in this study [36]. According to our MIMIC invariance models findings, it is possible to use the Bolivian Quechua PHQ-9 to perform comparisons between urban and rural residents, between men and women, age groups, marital status, and educational level.

We have identified some strengths and limitations in our study. This is the first cultural adaptation to Bolivian Quechua of the PHQ-9, a tool widely used to evaluate depressive symptoms. This implies the inclusion of an important Bolivian population, usually neglected, underserved or ignored in terms of mental health care. Since this is a written version, participants must have a minimum essential education (i.e., reading and writing skills), which is not always easy to find in the target population. Future studies must overcome this barrier by trying to reach all Quechua speakers using new communication technologies (e.g., apps with voice/language recognition). On the other hand, to estimate measurement invariance, we employed the Multiple-Indicators Multiple-Causes (MIMIC) due to its statistical power and not requiring proportional sample sizes to estimate invariance. However, unlike multigroup CFA, MIMIC models can only evaluate invariant intercepts and factor means. Consequently, we assume that the remaining structural and measurement parameters (e.g., factor loadings, error variance/covariance, factor variance/covariance) are equal across all levels of these variables.

This study is an essential step for improving public health actions (e.g., mental health services) for a historically underserved group. This version of the PHQ-9 can be gradually incorporated into national health surveys (e.g., Demographic and Health Surveys or INE in Bolivia), mental health programs (e.g., Municipal Comprehensive Legal Services or SLIM in Bolivia), as well as in community centres located in different regions of the country. Bolivian Quechua speakers will easily connect with a test expressing questions in the language they were born and lived in.

In conclusion, the PHQ-9 adapted to Bolivian Quechua offers a valid, reliable and invariant unidimensional measurement across groups according to the residence, sex, age, marital status and educational level. Comparisons can be made with the Bolivian Quechua PHQ-9 measurements through the mentioned groups.

## Data Availability

The data and complementary materials with which this study was developed are available at: https://doi.org/10.5281/zenodo.10075806.
